# The Utility of Pre-Procedural Blood Tests in Neuraxial Blocks: A Retrospective Study in High-Risk Patients

**DOI:** 10.3390/diagnostics14222588

**Published:** 2024-11-18

**Authors:** Sungho Moon, Daeseok Oh

**Affiliations:** Department of Anesthesia & Pain Medicine, Inje University Haeundae Paik Hospital, Busan 48108, Republic of Korea

**Keywords:** neuraxial blockade, blood tests, comorbidity, preoperative, pain management, glucose

## Abstract

**Background/Objectives**: The necessity and clinical utility of routine pre-procedural blood tests (PBTs) before neuraxial blockade remain controversial. This study evaluates the effectiveness of PBTs in identifying clinically significant conditions in an outpatient setting. **Methods**: This single-center retrospective study involved patients who received neuraxial blockades from January 2020 to August 2023. We extracted medical information and laboratory data from the electronic medical records during the pre-procedural period. Through a multivariate regression analysis, we identified patient factors associated with abnormal laboratory results. **Results**: Advanced age (OR, 1.021; *p* = 0.026) and a history of cancer (OR, 2.359; *p* = 0.016) were significantly associated with elevated CRP levels (>0.30 mg/dL). Severe hyperglycemia (≥200 mg/dL) was found in 24 patients (3.88%), with a history of cancer being a strong predictor (OR, 6.764; *p* < 0.001). No significant abnormalities were observed in PT or PTT. Reduced eGFR (<60 mL/min/1.73 m^2^) was detected in 8.62% of patients, despite no prior history of renal dysfunction. A multivariate analysis revealed that advanced age, hypertension, cancer, and coronary artery disease were significant predictors of abnormal PBT results, highlighting the importance of selective testing in high-risk patients. **Conclusions**: Routine PBTs are not universally required for all patients undergoing neuraxial blockade but can provide crucial information in high-risk populations. A selective testing approach based on individual risk factors is recommended to optimize patient safety and resource utilization. Future studies should aim to establish clear guidelines for targeted PBT use.

## 1. Introduction

Preoperative laboratory testing plays a critical role in diagnosing underlying conditions, assessing patient risk, and guiding perioperative management. However, routine testing in otherwise healthy individuals often provides limited clinical benefits and can lead to unnecessary healthcare costs and resource utilization. Despite guidelines advising against routine testing for ASA I or II patients undergoing low-risk, minimally invasive procedures, many clinicians continue to order these tests due to a lack of guideline awareness, concern about medico-legal issues, institutional mandates, and financial incentives [1,2]. While guidelines provide a framework for clinical practice, they do not replace the need for clinical judgment. A more effective approach would be to selectively conduct laboratory tests based on patient history, physical examination, and medical records, particularly when there are concerning findings or significant changes in the patient’s condition [1].

Neuraxial blockades are increasingly used as an effective treatment for pain management, particularly in the aging population with a high prevalence of degenerative diseases. While this procedure is generally safe and can be performed on an outpatient basis, it carries potential risks, such as bleeding, infection, and neurological complications, making pre-procedural evaluation crucial [3]. Unlike other minimally invasive procedures typically performed in outpatient settings, neuraxial blockades involve puncturing the spinal and epidural spaces, which, if complications arise, can lead to serious consequences, such as neurological damage. Despite the importance of pre-procedural blood tests (PBTs) in identifying risk factors and ensuring patient safety [4,5], there is a lack of clear evidence and guidelines on their use specifically for neuraxial procedures in outpatient settings. Current guidelines establish thresholds for platelet count, aPTT, and INR prior to performing neuraxial blocks, noting that while advanced hemostatic tests, such as thromboelastography, can aid in assessing bleeding risk, they remain insufficiently validated for neuraxial applications [4,5]. Other reviews have suggested that inflammatory markers, such as CRP and ESR, may help detect potential infections—especially spinal infections—and, although these markers have low specificity, they serve as early indicators that can aid in preventing complications [6]. The guidelines underscore the importance of PBTs for high-risk groups, including patients with coagulopathy, active infections, or significant comorbidities. However, there remains a lack of consensus regarding the need for routine PBTs in otherwise low-risk patients undergoing neuraxial procedures in outpatient settings. This ambiguity in clinical practice may lead to both overuse and underuse of PBTs, potentially impacting patient outcomes. This study aims to address this gap by evaluating the necessity and utility of routine PBTs in patients undergoing neuraxial blockades. We conducted a retrospective analysis of pre-procedural blood tests performed at our outpatient pain clinic from January 2020 to August 2023. Our objective is to determine whether these tests provide significant clinical value in predicting and preventing complications associated with neuraxial procedures and to identify patient factors that may be associated with abnormal test results.

Through this study, we seek to answer the following questions: Do routine PBTs offer substantial clinical value in the context of neuraxial blockade? Can specific patient factors predict abnormal test results that might alter clinical management? By addressing these questions, we hope to contribute to the development of more evidence-based guidelines for pre-procedural evaluation, ultimately reducing unnecessary testing and focusing resources on patients who would benefit most from targeted assessments. Our findings could play a crucial role in improving the safety and efficiency of neuraxial procedures.

## 2. Materials and Methods

### 2.1. Data Collection

After obtaining a waiver for the requirement of consent from our Institutional Review Board (HP IRB 2024-06-010), due to the retrospective nature of the study, we gathered data from the medical records of outpatients who visited a pain clinic between January 2020 and August 2023 and underwent blood tests for a neuraxial procedure. At our clinic, prior to conducting neuraxial procedures, blood tests are performed with patient consent to ensure the safety of the intervention. Neuraxial blocks are primarily administered to manage acute and chronic pain unresponsive to conservative treatments, with fluoroscopic guidance used to confirm spread patterns via contrast agents. Medications, including corticosteroids, are used unless contraindicated. The types of neuraxial blocks performed include interlaminar epidural block, transforaminal epidural block, selective nerve root block, facet joint block, medial branch block, and sympathetic block. We reviewed patient records obtained through an initial electronic search, which included patient demographics, comorbidities, and pain-specific information from our electronic medical records. The patient demographics encompassed age, gender, height, weight, body mass index (BMI), and the presence of comorbidities, such as a history of diabetes mellitus (DM), hypertension (HTN), coronary artery obstructive disease (CAOD) or arrhythmia, chronic renal insufficiency (CRI), or cancer. We excluded cases in which patients were considered cured after five years of cancer treatment from the comorbidity list. We recorded pain-specific information as the underlying diagnosis for neuraxial blockade, classifying the underlying diagnoses into categories, such as spinal disorders like stenosis and disc disease, based on their location, herpes zoster, postherpetic neuralgia, and other pain conditions. Postherpetic neuralgia was defined as pain persisting for more than three months following the rash onset. Pain intensity was measured using a numeric rating scale ranging from 0, indicating no pain, to 10, representing the worst imaginable pain. In this study, PBTs were identified based on blood work performed within two weeks prior to the procedure. The laboratory parameters collected included hemoglobin, white blood cell count (WBC), platelets (PLT), erythrocyte sedimentation rate (ESR), C-reactive protein (CRP), blood glucose, estimated glomerular filtration rate (eGFR), aspartate aminotransferase (AST), alanine aminotransferase (ALT), sodium, potassium, prothrombin time (PT), activated partial thromboplastin time (PTT), and international normalized ratio (INR). Patients with missing height and weight data were excluded to ensure consistency in calculating body mass index (BMI), a key factor in assessing metabolic and comorbid conditions. Abnormal results from the PBTs were identified, and factors associated with these abnormalities were determined.

### 2.2. Statistical Analysis

In Table 1, Table 2 and Table 3, continuous variables were represented as mean ± standard deviation. Categorical variables were expressed as frequencies (%). *t*-tests were applied to continuous variables that followed a normal distribution; otherwise, Mann–Whitney U tests were utilized. The Shapiro–Wilk test was used to assess the normality assumption of the distribution of continuous variables. For categorical variables, analyses were conducted using either the Pearson chi-squared test or Fisher’s exact test, depending on the data’s distribution. The variables included in the regression analysis were selected based on their completeness in the electronic medical records and their clinical relevance to the study objectives. Univariate logistic regression was first employed to assess the association between each independent variable and the outcome. Variables with *p* values < 0.05 in the univariate analysis were included in the multivariate logistic regression model. Multivariate logistic regression analysis was performed using the backward elimination method, considering all variables with a *p* value of <0.05 from the univariate analysis. This approach enabled the exclusion of non-significant variables while retaining those that contributed significantly to outcome prediction. SPSS software (version 25.0, SPSS Inc., Chicago, IL, USA) was used to perform these analyses.

## 3. Results

**Figure 1:**
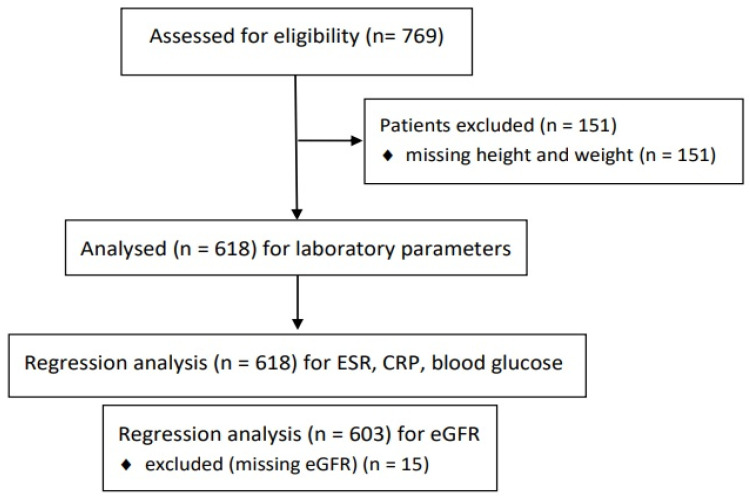
Flow diagram summarizing patient selection. ESR, erythrocyte sedimentation rate; CRP, C-reactive protein; eGFR, estimated glomerular filtration rate.

In total, 769 patients were initially selected. Initially, 151 patients lacked height and weight data, resulting in 618 cases of patients who had received PBTs (Figure 1). Table 1 displays patient characteristics and clinical data. Table 2 provides a summary of cases that showed abnormal findings in blood tests. Ten patients were confirmed to have additional diagnoses related to abnormal results identified during the follow-up period (Table 3).

### 3.1. WBC, ESR, CRP

Forty-three patients exhibited WBC > 10.0 × 10^9^/L. Seventy-six patients showed an ESR increase exceeding 20 mm/h. Ninety-three patients presented with CRP levels beyond 0.30 mg/dL. No significant difference was observed among the groups with elevated WBC. In cases involving ESR and CRP, predictive factors associated with elevated levels were identified through multivariate regression analysis. Advanced age (*p* = 0.001) and a history of CRI (*p* = 0.038) correlated significantly with increased ESR (>20 mm/h). Advanced age (*p* = 0.026) and a history of cancer (*p* = 0.016) were significantly correlated with heightened CRP levels (>0.30 mg/dL) (Table 4).

There was a single case of infectious spondylodiscitis identified early based on inflammatory markers. A 47-year-old male with pain radiating to the upper limb presented for a cervical injection. A CT scan conducted two days before his visit showed foraminal stenosis, and laboratory tests indicated leukocytosis (12.95 × 10^9^/L) and elevated CRP (2.08 mg/dL). Subsequent tests on the day of his visit confirmed an escalation in inflammatory markers (Table 3). The patient had no underlying medical conditions but had undergone an invasive neck procedure at another clinic one week prior. An MRI scan confirmed infectious spondylodiscitis with phlegmon and abscess formation. Methicillin-resistant Staphylococcus aureus was isolated from the blood cultures. The patient recovered following antibiotic treatment. Additionally, in some cases, procedures were deferred due to elevated inflammatory markers, with further diagnoses made during follow-up. For instance, an 82-year-old male, who had been previously treated for herpes zoster, presented with persistent chest pain despite ongoing oral medication, reporting an NRS score of 90. A thoracic epidural block was planned, but pre-procedural blood tests revealed elevated CRP and ESR levels (Table 3). Although the physical examination was unremarkable, the patient received IV therapy and was discharged for observation. Two days later, he returned with worsening chest pain and shortness of breath. An emergency department evaluation led to a diagnosis of pneumonia, and he was treated with antibiotics. Additional cases diagnosed during follow-up included non-tuberculous mycobacterium infection, polymyalgia rheumatica, and acute pyelonephritis.

### 3.2. Glucose

Out of a total of 618 individuals, 96 were identified with high random blood glucose (≥140 mg/dL). Among these, 51 had no history of DM. Remarkably, 24 (3.88%) exhibited glucose levels of ≥200 mg/dL, of whom eight had no prior diagnosis of DM, while sixteen were receiving treatment for known DM. A multivariate regression analysis revealed that a history of cancer (*p* < 0.001) was significantly associated with severe hyperglycemia (≥200 mg/dL), alongside DM history (*p* < 0.001) (Table 4). In univariable analyses, a history of cancer was associated with elevated blood glucose (≥140 mg/dL), although it was not significant in multivariate analyses (*p* = 0.052). Additionally, two cases of previously unrecognized DM were diagnosed with diabetic neuropathy following incidentally discovered hyperglycemia (Table 3).

### 3.3. PLT, PT/PTT

Of the 618 individuals, 5 presented with PLT levels that decreased to below 100 × 10^9^/L. One patient with a count of 51 × 10^9^/L had liver cirrhosis; additionally, among the other four with PLT counts ranging between 70 and 100 × 10^9^/L, two had a history of liver disease (cirrhosis, had undergone liver transplantation). There were no significant findings or medical histories in the other cases, and other PBTs were unremarkable. No cases of abnormal INR or significant deviations in PT and aPTT were noted in patients without hereditary bleeding disorders or prior bleeding events (Table 2).

### 3.4. eGFR

Out of 618 patients, 603 were analyzed after excluding 15 patients with missing eGFR. A total of 59 patients exhibited decreased eGFR (<60 mL/min/1.73 m^2^), among whom 52 (88.14%) had no history of CRI. The multivariate regression analysis demonstrated that variables such as advanced age (*p* < 0.001), history of HTN (*p* = 0.012), cancer (*p* = 0.019), and CAOD or arrhythmia (*p* < 0.001) significantly predicted decreased eGFR (Table 4). Three patients were identified with severe kidney function loss (eGFR < 30 mL/min/1.73 m^2^): a 64-year-old male and an 85-year-old female with no previous renal insufficiency (Table 3) and another patient (eGFR = 11 mL/min/1.73 m^2^) who had been previously treated for HTN and CRI. The 64-year-old male, although a unique case, was transferred to the emergency room due to severe unexpected hyperkalemia, where he was diagnosed with acute kidney injury due to chronic renal disease. After treatment, he recovered and was discharged but is currently undergoing dialysis. (Table 3).

**Table 1:** Baseline patient characteristics.

Total No. of Patients	*N* = 618
Age (years), mean	63.78 ± 13.45
BMI	24.18 ± 3.26
Sex, M/F (%)	288/330 (46.6%)
Diagnosis for blocks	
Spine pathology, *n* (%)	473 (76.5%)
HZ and PHN, *n* (%)	113 (18.3%)
Others, *n* (%)	32 (5.2%)
NRS	62.15 ± 18.26
Comorbidities	
None, *n* (%)	288 (46.6%)
HTN, *n* (%)	262 (42.4%)
DM, *n* (%)	96 (15.5%)
CAOD or arrhythmia, *n* (%)	65 (10.5%)
Cancer, *n* (%)	44 (7.1%)
CRI, *n* (%)	10 (1.6%)

Values are expressed either as mean ± standard deviation or as an absolute number (percentage). BMI, body mass index; M, male; F, female; The abbreviations NRS, HZ, PHN, DM, HTN, CAOD, and CRI represent numerical rating scale, herpes zoster, postherpetic neuralgia diabetes mellitus, hypertension, coronary artery obstructive disease, and chronic renal insufficiency, respectively.

**Table 2:** Routine laboratory testing and abnormal results before performing neuraxial blockade (*n* = 618).

Test Type (Normal Value)	Number of Abnormal Results
Hemoglobin (13.0~17.0 g/dL)	
<13.0	147
White blood cell (4.0~10.0 × 10^9^/L)	
>10	43
Platelet (140~440 × 10^9^/L)	
70~100	4
<70	1
ESR (0~20 mm/h)	
>20	76
CRP (<0.30 mg/dL)	
>0.3	93
Glucose (70~110 mg/dL)	
≥140	96
≥200	25
eGFR (>90 mL/min/1.73 m^2^)	
<60	59 ^a^
<30	3 ^a^
AST (~40 U/L)	
>40	39
ALT (~41 U/L)	
>41	80
Sodium (136~145 mmol/L)	
>145	5
Potassium (3.5~5.1 mmol/L)	
≥5.5	8
INR (0.80~1.20%)	
1.2~1.4	6
>1.4	0

Values are presented as absolute numbers. ^a^ (*n* = 608) patients. ESR, erythrocyte sedimentation rate; CRP, C-reactive protein; eGFR, estimated glomerular filtration rate; AST, aspartate aminotransferase; ALT, alanine aminotransferase; INR, international normalized ratio.

**Table 3:** Diagnosis confirmed after further evaluation following abnormal laboratory results.

	Past History	WBC	ESR	CRP	eGFR	AST	ALT	Glucose	Na	K	Diagnosis	Additional Diagnosis
M/82	None	6.88	20	10.20							PHN	Pneumonia
F/56	Cancer	11.7	50	9.04							S	Non-tuberculous mycobacterium
M/84	HTN DM	10.34	43	6.27	55						S	Polymyalgia rheumatica
F/72	HTN CAOD CRI	11.21	86	5.31	50						S	Acute pyelonephritis
M/47	None	11.47	36	13.23							S	Infectious spondylitis
F/55	None					152	301				HZ	Drug induced hepatitis
M/52	None							452	134		S	Diabetic neuropathy
M/39	None							504	129		S	Diabetic neuropathy
M/64 F/85	HTN DM HTN DM CAOD		26		12 27			164	135	8.2	S S	CRF CRI

Values are expressed as blood test results without units. CRI is defined as an eGFR of <60 mL/min/1.73 m^2^. CRF is defined as an eGFR of <15 mL/min/1.73 m^2^. M, male; F, female; DM, diabetes mellitus; HTN, hypertension; CAOD, coronary artery obstructive disease; CRF, chronic renal failure; CRI, chronic renal insufficiency; WBC, white blood cell (10^9^/L), ESR, erythrocyte sedimentation rate (mm/h); CRP, C-reactive protein (mg/dL); eGFR, estimated glomerular filtration rate (mL/min/1.73 m^2^); AST, aspartate aminotransferase (U/L); ALT, alanine aminotransferase (U/L); Na, sodium (mmol/L); K, potassium (mmol/L); PHN, postherpetic neuralgia; HZ, herpes zoster; S, spine pathology.

**Table 4:** Summary of multivariate logistic regression analysis of factors predicting abnormal results on pre-procedure blood testing.

	ESR (>20 mm/h)		CRP (>0.30 mg/dL)		Glucose (≥200 mg/dL)		eGFR (<60 mL/min/1.73 m^2^)	
	OR	95% CI	OR	95% CI	OR	95% CI	OR	95% CI
Advanced age	1.037	1.015–1.059	1.021	1.002–1.040			1.101	1.067–1.136
Comorbidities								
HTN							2.153	1.188–3.905
DM					14.352	5.446–37.825		
CAOD or arrhythmia							3.306	1.751–6.230
Cancer			2.359	1.174–4.739	6.764	2.261–20.213	2.917	1.194–7.122
CRI	4.013	1.083–14.875					*	*

Estimated odds ratio (OR). HTN, hypertension; DM, diabetes mellitus; CAOD, coronary artery obstructive disease; CRI, chronic renal insufficiency; ESR, erythrocyte sedimentation rate; CRP, C-reactive protein; eGFR, estimated glomerular filtration rate; CI, confidence interval. * Known CRI is excluded as a variable in the analysis for eGFR.

## 4. Discussion

The primary contraindications for neuraxial procedures include sepsis, fever, viral infections, coagulopathy, and preexisting central nervous system disorders [7]. The rationale for performing PBTs before neuraxial blockade is to detect systemic illnesses, assess coagulation status, evaluate thrombocytopenia, and check renal function, thereby ensuring safe and effective analgesia by identifying potential risk factors. We present significant findings from PBTs based on retrospective results. This population-based study of outpatients suggests that routine PBTs may not be necessary for all patients undergoing neuraxial blockade; however, they can offer significant clinical value in specific high-risk populations. For instance, patients with advanced age, a history of cancer, or chronic comorbidities such as diabetes or renal dysfunction may benefit from selective testing. In these groups, abnormalities in inflammatory markers, such as ESR and CRP, helped identify previously undiagnosed infections, while abnormal glucose levels led to the diagnosis of undiagnosed diabetes. These findings suggest that selective PBTs based on individual risk factors can improve patient safety by optimizing pre-procedural planning and reducing complications. This study further indicates that specific patient factors, such as advanced age, history of cancer, and diabetes, are significantly associated with abnormal blood test results, with direct implications for clinical management. Elevated ESR and CRP levels were more common in patients with advanced age or a history of cancer, indicating a higher risk of infection or inflammatory conditions. Similarly, patients with diabetes often presented with hyperglycemia, necessitating careful glucose monitoring, especially following steroid injections. These findings underscore the importance of customizing PBTs according to individual patient characteristics to enhance safety and minimize unnecessary testing.

Markers such as CRP and eGFR provided valuable insights into undiagnosed conditions that might otherwise go unnoticed during routine clinical assessments. In some cases, elevated CRP levels led to the diagnosis of serious infections, such as pneumonia or spondylodiscitis, while decreased eGFR levels prompted additional nephrology consultations and modifications in medication management. This reinforces the value of selective PBTs for high-risk individuals. Routine PBTs for all patients, however, are not clinically warranted, and a selective approach should be prioritized based on the patient’s health profile and risk factors.

### 4.1. WBC, ESR, CRP

Fulminant sepsis or massive site infection are absolute contraindications against neuraxial blockade [7]. Spinal infection can occur by entry into the epidural space from a contiguous infection (e.g., psoas abscess, osteomyelitis, skin infection), hematogenous spread, or through direct inoculation (e.g., steroid injection, surgery, nerve block, acupuncture) [6]. Hematogenous spread in bacteremic patients during spinal needle insertion can lead to bleeding into the subarachnoid space, thereby increasing the risk of meningitis [8]. Experts have advised against performing neuraxial procedures in patients with untreated systemic infections [9]. However, the level of systemic infection that constitutes a risk factor for infection via hematogenous spread remains undefined. Several markers are considered crucial for diagnosing infectious diseases [10]. Infections may present with leukocytosis and elevated ESR and CRP levels [6]. However, the limited sensitivity of the WBC count makes it the least informative [6]. ESR is a sensitive marker, but it has low specificity. A CRP level can rise dramatically in response to injury, infection, and inflammation, serving both as a marker and an active participant in the inflammatory process [11]. In previous research, infection was identified as the most frequent cause of elevated CRP levels (55.1%), followed by rheumatologic diseases, other inflammatory conditions, and malignancy [12]. Specially, CRP is a reliable indicator of spinal infections [6]. Although there are limitations in diagnosing infectious diseases based solely on blood test results, meticulous evaluation—such as tracking changes through additional tests or other thorough assessments—is often necessary when abnormalities are detected. In our clinical practice, when elevated inflammatory markers are detected, the standard protocol is to postpone neuraxial block procedures and perform additional investigations, including a review of recent invasive procedures, medical history, and physical examination to identify potential sources of infection. In our study, various infectious disorders were identified through routine PBTs (Table 3). Infectious diseases often accompany pain syndromes: cellulitis near prosthetic leg in stump pain, diabetic foot in diabetic neuropathy, pneumonia in elderly patients, herpes zoster in immunocompromised patients, and septic arthritis or spondylodiscitis in degenerative disorders. As interventional pain management grows, the surgical site infection risk increases [13]. All identified diseases based on infection-related markers were not considered absolute contraindications for neuraxial blockades, yet the findings provided unexpected clinical insights that could guide the diagnosis and management of patients before procedures.

### 4.2. Glucose

Preoperative blood glucose testing is considered reasonable in diabetic patients, obese patients, and those on long-term steroid therapy because there is a correlation between elevated blood glucose and postoperative poor outcomes [14]. Specifically, elevated blood glucose levels have been associated with an increased risk of infections among patients scheduled for various orthopedic surgery [1]. Maintaining perioperative blood glucose levels below 200 mg/dL is crucial in preventing surgical site infections, regardless of diabetes status, as supported by strong recommendations [10]. Therefore, pre-procedural glucose testing might be of critical importance, as the elderly population might also be prone to undiagnosed DM.

The most common side effect of steroids used in neuraxial analgesia is elevated blood glucose. Hyperglycemia after epidural injection has been observed in patients both with and without DM [15], although this glucose elevation has been shown to persist longer in DM than in non-DM patients [16]. DM patients experience pronounced systemic effects from glucocorticoids due to diminished clearance and extended exposure [15]. This typically results in an average increase of 125.96 ± 100.97 mg/dL in blood glucose after an epidural injection [17]. Therefore, DM patients can reasonably expect a temporary elevation of blood glucose by 100 mg/dL or more for approximately 48 to 72 h following an epidural steroid injection [17]. This response also depends on the steroid dose used [18]. It is advisable to exercise caution when administering a neuraxial steroid injection to patients with DM, and frequent glucose monitoring is highly recommended [19]. If not assessed beforehand, patients with unrecognized hyperglycemia might experience reactions similar to those observed in diabetic patients after steroid injection.

In our study, some patients were not diagnosed with DM but exhibited confirmed hyperglycemia, and there were patients who, while undergoing treatment for DM, were confirmed to have elevated glucose levels. Notably, those with a history of cancer were found to have a risk of elevated blood glucose. Although the underlying mechanisms vary and require further investigation, the relationship between hyperglycemia and tumor development has been previously documented [20]. Patients who are candidates for neuraxial steroid injection should be informed that an increase in blood glucose may occur post-procedure [17]. At our institution, when hyperglycemia is detected, we consult with the patient to determine whether to delay the procedure or proceed with the neuraxial block without steroid administration. While there is no conclusive evidence regarding the systemic effects of transient hyperglycemia, awareness of a patient’s blood glucose levels before the procedure can help reduce steroid use and related complications such as hyperglycemia and infection. In certain cases, effective pain control through neuraxial blockade can be achieved without steroid use [21]. Clinicians should also recognize that, whether in cases of treated DM or undiagnosed DM, well-controlled blood glucose levels cannot always be ensured.

### 4.3. PLT, PT/PTT

A systematic review indicates that routine coagulation tests, such as PT and PTT, are not effective in predicting postoperative bleeding risk in unselected patients and are therefore not recommended [1]. These preoperative coagulation studies should be reserved for patients taking anticoagulants, those with a history of bleeding abnormalities, or those with conditions that predispose them to coagulopathy, such as liver disease or malnutrition.

Neuraxial blockade inherently carries some risk of inducing bleeding, especially in patients with preexisting bleeding diatheses (e.g., thrombocytopenia, coagulation factor deficiencies, renal dysfunction) or those with medically induced coagulation dysfunction. Although bleeding complications are extremely rare with neuraxial procedures, certain bleeding types can lead to severe complications [22]. Specifically, bleeding within the vertebral column has the potential to cause permanent paralysis if it is not managed within 8 to 12 h [22,23]. This concern may drive some physicians to routinely order coagulation screening prior to performing a neuraxial blockade, although the efficacy and cost-effectiveness of this practice are debatable from the perspectives of evidence-based medicine. The American Society of Regional Anesthesia recommends evaluating the need for coagulation tests (complete blood count, PT, PTT) individually for each patient [24]. In our study, no patients exhibited abnormal PT or PTT that affected the procedure, even those with comorbidities. PT and PTT screening in low-risk patients was deemed unnecessary. A PLT of <100 × 10^9^/L is generally considered a risk factor for spinal hematoma, but when there are indications for a neuraxial blockade, the risks and benefits to the patient must be weighed [25]. If the PLT is ≥70 × 10^9^/L, there is likely to be a low risk of neuraxial hematoma, allowing for a neuraxial procedure if clinically indicated [4]. Our results also indicate that patients without underlying disease may have hematologic and coagulation indices within an acceptable range for performing neuraxial blockade. Conducting routine coagulation studies before the blockade may be beneficial if a patient currently has a history of taking anticoagulants (warfarin), suffers from coagulopathy, or has chronic liver disease [1]. Specifically, hepatic failure results in factor deficiencies and thrombocytopenia [26].

### 4.4. eGFR

Kidney function is widely recognized as a crucial determinant of prognosis in various surgical settings [1,27]. Several factors underscore the necessity of assessing renal function before administering neuraxial blockade. Firstly, renal dysfunction is identified as a risk factor for vertebral hematoma, along with preexisting coagulopathy, the use of anticoagulation medications, aging, gender, spinal abnormalities, the use of larger needles, and multiple needle attempts [23]. Uremia is linked to spontaneous hemorrhage, partly due to PLT dysfunction [5]. Moreover, renal impairment can alter the metabolism and clearance of drugs, particularly those excreted by the kidneys, such as platelet inhibitors and anticoagulants. Patients with moderate renal impairment encounter an increased risk of spontaneous hemorrhage due to delayed excretion of antihemostatic drugs [5]. Therefore, extended discontinuation times are recommended before neuraxial blockade. Spinal hematomas have occurred in individuals taking antiplatelet agents, despite adherence to discontinuation protocols [28]. Consequently, renal function could be considered a critical parameter when evaluating a patient’s bleeding risk. Secondly, contrast media are used both to verify accurate tissue targeting and confirm the exclusion of flow to non-target tissues in various neuraxial procedures. Assessing renal function beforehand is essential to prevent contrast-induced nephropathy [29]. Furthermore, although indirectly related to neuraxial blockade, measuring renal function provides valuable insights that can inform the appropriate dosages of gabapentinoids, which are commonly used as first-line treatments for neuropathic pain in pain clinics. Evaluating renal function might influence the selection and dosage of these medications. In cases of decreased eGFR, medication adjustments may be necessary, and minimizing needle trauma is important. Platelet function testing may also be useful in predicting bleeding tendencies when needed.

eGFR is known to be effective as a predictor of renal dysfunction [1] and serves as a reliable test for detecting subclinical renal failure [27]. Low eGFR is associated with clinical risk factors, such as DM, HTN, aging, malignancy, and obesity, yet fewer than 5% of early CRI patients are aware of their condition [30]. It is also noted that many patients with decreased eGFR demonstrated no prior history of renal dysfunction in this study.

Our study has several limitations. As a cross-sectional analysis, it does not assess the adverse effects of neuraxial blockade in patients with abnormal primary blood tests. Various cases involved additional diagnoses through follow-up tests, resulting in discontinued or improved outcomes. We did not evaluate whether abnormal test results influenced complications or outcomes. Clinical management was adjusted based on the findings, including postponing procedures, further evaluations, or modifying the injectate, as guided by clinical judgment. However, factors such as non-adherence to follow-up recommendations or loss to follow-up, particularly in outpatient settings, make it challenging to provide comprehensive quantitative data. The absence of established guidelines makes proceeding with abnormal results controversial, necessitating large-scale studies to clarify the associated benefits and risks. Moreover, preexisting medical information may not adequately explain a patient’s condition in outpatient settings. PBTs can selectively provide valuable predictive information. The retrospective, single-center design of our study may limit the generalizability of the results, and the relatively small sample size could introduce statistical bias, especially in subgroup analyses. Inherent selection biases are present, as patient data were limited to those available in electronic medical records. Patient histories are often self-reported, reducing reliability, and we did not explore comorbidities beyond medical records. These factors may limit the generalizability of our results to a broader patient population. Additionally, the lack of external validation restricts the applicability of the findings in other clinical settings. Prospective studies with validation are needed to confirm these findings and guide future practice. Although patients on continuous steroid therapy were excluded, recent steroid injections were not tracked, which may have influenced outcomes. Some variables, such as anticoagulant use and the timing of lab tests, were also missing, and no corrections for multiple testing or interactions were applied. The timing of blood tests may not align perfectly with procedures, especially in cases in which glucose levels are influenced by meals, as we used random plasma glucose levels.

## 5. Conclusions

PBTs can provide valuable clinical information that may not be identified through patient informatics and comorbidities prior to performing neuraxial blockade in an outpatient setting. Patient medical comorbidities can serve as a reference for selective blood test screening, although they may not fully account for the patient’s condition. Our study emphasizes the clinical utility of PBTs in high-risk populations, showing that targeted testing based on individual patient characteristics can enhance pre-procedural planning and improve patient safety. Routine testing for all patients is not warranted; however, selective PBTs, particularly for older patients or those with significant comorbidities, can assist in detecting undiagnosed conditions and guide clinical management more effectively.

## Data Availability

The original contributions presented in the study are included in the article; further inquiries can be directed to the corresponding author.
